# Smartphone-Compatible Colorimetric Detection of CA19-9 Using Melanin Nanoparticles and Deep Learning

**DOI:** 10.3390/bios15080507

**Published:** 2025-08-05

**Authors:** Turgut Karademir, Gizem Kaleli-Can, Başak Esin Köktürk-Güzel

**Affiliations:** 1 Department of Electrical and Electronics Engineering, Faculty of Engineering, Izmir Demokrasi University, 35140 Izmir, Türkiye; turgut.karademir@idu.edu.tr; 2 Department of Biomedical Engineering, Faculty of Engineering, Izmir Demokrasi University, 35140 Izmir, Türkiye; gizem.kalelican@idu.edu.tr

**Keywords:** melanin nanoparticle, colorimetric analysis, image segmentation, paper-based analytical devices (PADs), regression models, U-NET, dedectron 2, YOLOv8

## Abstract

Paper-based colorimetric biosensors represent a promising class of low-cost diagnostic tools that do not require external instrumentation. However, their broader applicability is limited by the environmental concerns associated with conventional metal-based nanomaterials and the subjectivity of visual interpretation. To address these challenges, this study introduces a proof-of-concept platform—using CA19-9 as a model biomarker—that integrates naturally derived melanin nanoparticles (MNPs) with machine learning-based image analysis to enable environmentally sustainable and analytically robust colorimetric quantification. Upon target binding, MNPs induce a concentration-dependent color transition from yellow to brown. This visual signal was quantified using a machine learning pipeline incorporating automated region segmentation and regression modeling. Sensor areas were segmented using three different algorithms, with the U-Net model achieving the highest accuracy (average IoU: 0.9025 ± 0.0392). Features extracted from segmented regions were used to train seven regression models, among which XGBoost performed best, yielding a Mean Absolute Percentage Error (MAPE) of 17%. Although reduced sensitivity was observed at higher analyte concentrations due to sensor saturation, the model showed strong predictive accuracy at lower concentrations, which are especially challenging for visual interpretation. This approach enables accurate, reproducible, and objective quantification of colorimetric signals, thereby offering a sustainable and scalable alternative for point-of-care diagnostic applications.

## 1. Introduction

Paper-based analytical devices (PADs) have emerged as cost-effective, portable, and user-friendly platforms for diagnostics, utilizing capillary action for fluid transport without the need for external power [1,2]. These characteristics make PADs highly suitable for point-of-care applications and use in low-resource environments. Their widespread use in environmental monitoring, food safety, and medical diagnostics is attributed to their low production cost, rapid response time, and minimal consumption of reagents [3,4]. Recent advances have integrated nanomaterials into PAD architectures, significantly improving their sensitivity, specificity, and stability [5,6]. Among these, gold nanoparticles (AuNPs) have been widely employed due to their unique localized surface plasmon resonance (LSPR), which produces a vivid red-to-blue color change upon aggregation, facilitating straightforward visual detection [7,8,9].

Despite their superior optical characteristics and widespread use in biosensing, the long-term sustainability of gold nanoparticles (AuNPs) is increasingly challenged due to their high cost, limited natural availability, and growing environmental concerns [10,11]. Between 2019 and 2024, over 71,000 Scopus-indexed publications have focused on AuNPs, reflecting their popularity, which is driven by properties such as high surface-to-volume ratio, electrical conductivity, chemical stability, and LSPR-mediated colorimetric response—features that enable efficient signal transduction and tunable surface functionalization [12]. However, the broad application of AuNPs raises significant ecological and toxicological concerns. At the nanoscale, their enhanced bioavailability promotes unintended accumulation in vital organs including the brain, liver, and kidneys. Particularly alarming is their demonstrated ability to cross biological barriers, such as the blood–brain barrier, and persist in neural tissues, contributing to long-term neurotoxicity [13,14]. Factors such as particle size, surface charge, and electrostatic state further govern their biodistribution and clearance kinetics [15]. Experimental studies have shown that intraperitoneally administered AuNPs accumulate in the hippocampus and induce cognitive dysfunction via monoaminergic dysregulation. Notably, pre-administration of tea-derived melanin nanoparticles (MNPs) effectively prevented hippocampal AuNP deposition and preserved cognitive performance by stabilizing monoamine levels and maintaining neuronal integrity [16]. These findings underscore the urgency of developing biocompatible, non-accumulative nanomaterials for diagnostic use. In this regard, MNPs derived from *Sepia officinalis* ink offer a biodegradable and metal-free alternative, combining broad-spectrum optical absorbance with abundant functional groups suitable for bioconjugation. Importantly, previous studies have also demonstrated that Sepia-derived MNPs exhibit high biocompatibility, with negligible cytotoxicity reported across multiple healthy cell lines (e.g., HUVEC, NIH 3T3, and L929) even at concentrations exceeding 100 µg/mL [17,18]. This reinforces their suitability for biosensing applications where long-term biostability and safety are critical [19,20,21,22,23]. Although MNPs eliminate the toxicological risks associated with metal-based systems, their inherently subtle yellow-to-brown chromatic shift requires external analytical assistance for reliable signal interpretation [24,25].

To overcome these limitations, we present a proof-of-concept platform that integrates MNP-based colorimetric sensing with artificial intelligence (AI)-assisted image processing for reliable and instrument-free quantification. In this study, MNPs were functionalized with glutaraldehyde-activated secondary amine groups and conjugated to anti-CA19-9 antibodies, allowing selective binding to the CA19-9 model biomarker for pancreatic cancer. CA19-9 was selected as a proof-of-concept biomarker due to its established clinical relevance and routine use in the management of pancreatic adenocarcinoma [26]. Elevated serum levels of CA19-9 are observed in approximately 80–90% of pancreatic cancer patients at the time of diagnosis, making it the most widely adopted tumor marker for disease progression monitoring and treatment response evaluation [27]. Although it is not suitable as a general population screening biomarker due to limited specificity in early-stage disease, the development of low-cost and repeatable platforms such as PADs could improve its utility in high-risk populations or resource-limited contexts [28]. Furthermore, CA19-9’s glycoprotein structure enables stable immobilization and selective recognition on nanoparticle-modified surfaces, making it technically well-suited for evaluating the analytical performance of new biosensing approaches. Upon antigen interaction, a color change occurs in the paper-based sensor; however, due to the subtlety of the melanin-induced optical shift, conventional visual assessment is insufficient for robust quantification.

Studies aiming to detect color changes in colorimetric biosensors and extract information using image processing typically involve manually selecting the Region of Interest (ROI) and calculating the intensity ratios of specific color channels within this manually selected area, or converting these color channels to other color spaces (HSV, CIELab*, etc.) for measurement. For example, the hue channel’s sensitivity to refractive index changes can enhance the sensitivity of colorimetric sensors, allowing more precise measurements. Additionally, the saturation channel’s ability to mitigate the effects of ambient light ensures accurate measurements under varying conditions. The CIELab* color space, being closely aligned with human visual perception, is widely used in image processing applications. The choice and design of the color space depend on the specific requirements of the experiment. While early studies such as Nguyen et al. [29] utilized manually selected ROIs and simple channel ratios (e.g., green-to-red intensity) for colorimetric biosensing, more recent research has explored automated approaches. For example, Phuangsaijai et al. [30] evaluated the predictive performance of multiple color spaces (RGB, CMYK, HSV, and CIELab*) combined with various preprocessing strategies to estimate water quality parameters from colorimetric sensor strips. Despite their automation attempts, such studies generally focus on specific experimental settings and often require controlled image capture environments or predefined sensor layouts.

In contrast, our study leverages melanin-based paper biosensors for the non-invasive detection of CA19-9 and introduces a fully automated image analysis pipeline. Unlike prior work, our system can reliably process images captured from arbitrary angles or lighting conditions, eliminating the need for a fixed experimental setup. This is achieved through machine learning models that segment the sensor area and normalize lighting and orientation artifacts using a control blank. Recent advances in machine learning have significantly reduced the cost of analysis and enabled more flexible, user-friendly solutions [31]. By integrating these advancements into a web-based analysis tool, our framework facilitates scalable and accurate biosensor usage without requiring expert intervention or standardized imaging environments.

**Figure 1:**
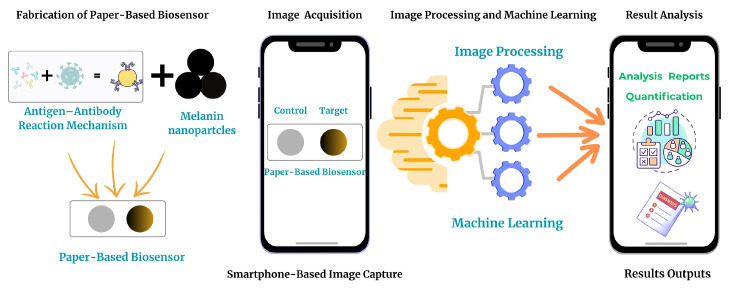
Schematic overview of the proposed biosensor workflow. Initially, a paper-based biosensor is fabricated using melanin nanoparticles and an antigen–antibody interaction mechanism. Upon sample application, the target zone undergoes a visible color change depending on the biomarker concentration, while the control zone remains unchanged. The biosensor image is then captured using a smartphone. Subsequently, machine learning and image processing algorithms are employed to analyze the image. Finally, the quantified results, such as biomarker concentration, are displayed on the smartphone interface for rapid and user-friendly evaluation.

To operationalize this framework in real-world settings, we developed a smartphone-compatible imaging pipeline that employs deep learning for automatic Region of Interest (ROI) segmentation and machine learning regression models, including XGBoost and gradient boosting, for precise estimation of analyte concentration. As illustrated in Figure 1, this approach eliminates the need for manual ROI selection, which is commonly required in conventional smartphone-based image analysis workflows, and mitigates the impact of lighting variability and user subjectivity. Our AI-enhanced system enables real-time, portable, and accurate colorimetric interpretation using only a mobile device, providing a cost-effective and scalable solution for biosensor readout. This work demonstrates the synergistic combination of green nanomaterials and intelligent computation for next-generation biosensing. By addressing both the environmental concerns associated with traditional plasmonic systems that rely primarily on gold nanoparticles and the interpretive limitations of human vision, our method lays the foundation for the development of sustainable, accessible, and standardized diagnostic technologies in decentralized settings.

## 2. Materials and Methods

### 2.1. Melanin Nanoparticles Extraction and Purification

Natural melanin nanoparticles (MNPs) were extracted from commercially available *Sepia officinalis* ink following the method outlined by Eom et al. [32]. The process began by diluting cuttlefish ink (*Sepia officinalis*) and centrifuging it at 10,000 rpm for 20 min. After the supernatant was removed, the pellet was rinsed with deionized water (DIW) to remove salts and impurities, with the process repeated three times. The purified pellets were then dried in an oven at 50 °C for 2 days [32]. A stock MNP solution was prepared by dispersing measured amounts of the dried powder in DIW and sonicating it overnight in a bath-type sonicator before use.

### 2.2. Fabrication of Anti-CA 19-9 Doped MNPs Functionalized Paper Analytical Devices

Surface activation of MNPs was performed using an aqueous glutaraldehyde solution (Sigma-Aldrich, St. Louis, MO, USA), a widely used bifunctional cross-linking agent in biosensor applications. For covalent functionalization, MNP suspensions (5% *w/v*) were incubated with 25% (*v/v* in DIW) glutaraldehyde under gentle agitation at room temperature for 2 h, enabling the formation of covalent bonds between the aldehyde groups of glutaraldehyde and secondary amine groups on the melanin surface. Following activation, excess glutaraldehyde was removed by centrifugation at 10,000 rpm for 20 min. The resulting pellet was washed three times with deionized water under identical conditions to eliminate unbound reagent. The activated MNPs were then air-dried at 50 °C for 48 h and resuspended in ultrapure water using bath sonication. For antibody immobilization, 100 µL of anti-CA 19-9 (2 µg/mL) was added to the glutaraldehyde-activated MNP suspension and stirred magnetically at room temperature for 24 h. The antibody concentration was selected based on our previous findings, where 2 µg/mL was determined to be the optimal concentration for maximizing biosensor response without inducing surface saturation [33]. Covalent conjugation was achieved via Schiff base formation between the primary amines of the antibody and the aldehyde functionalities on the nanoparticle surface. To prevent nonspecific interactions, the remaining unreacted aldehyde groups were blocked by incubation with 1% (*w/v*) bovine serum albumin (BSA) (Acros Organics, Morris Plains, NJ, USA) for 1 h. Finally, the functionalized nanoparticles were washed, centrifuged, and dried for subsequent integration into paper-based analytical devices [33,34,35].

To fabricate the colorimetric detection zone, anti-CA19-9-functionalized MNPs were dissolved in DIW and dropped onto predefined regions of the PADs via a drop-casting technique. Samples were dried overnight at room temperature to ensure firm adhesion of nanoparticles onto the nitrocellulose network. As a control group, unmodified paper substrates without melanin or antibody modification were prepared under the same conditions to enable baseline correction and contrast differentiation during image acquisition and analysis. Importantly, this study did not aim to investigate the direct interaction between CA19-9 (LifeSpan BioSciences, Inc., Seattle, WA, USA) and its antibody under flow-based or dynamic conditions. Rather, the primary objective was to examine whether differences in MNP concentrations, when immobilized in the test zones, could yield distinguishable color intensities that are trackable via digital image processing. Although the antibody immobilization step was included, it was not intended to facilitate actual biomarker binding in this study. Instead, it was incorporated to simulate the surface chemistry expected in future biosensing applications and to evaluate how the presence of antibodies might influence the chromatic characteristics of melanin.

This strategic approach was adopted because future iterations of the platform will involve the real-time detection of CA 19-9 in fluidic systems, where anti-CA19-9-decorated MNPs will interact with the analyte, and melanin signal changes will be monitored accordingly. Therefore, the current work focuses exclusively on validating whether nanoparticle concentration can modulate measurable color intensity in a controlled and quantifiable manner, thereby establishing a critical foundation for downstream bioassay design.

This initial validation confirms that melanin-based nanoparticles produce measurable color changes. As a next step, we aim to accurately identify the sensor regions in the images, which is crucial for subsequent stages of analysis.

**Figure 2:**
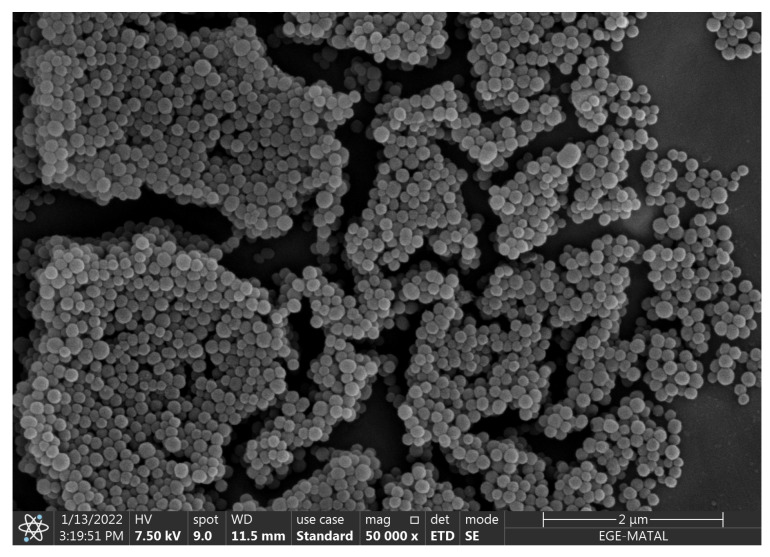
Scanning Electron Microscopy (SEM) image of melanin nanoparticles (MNPs) synthesized for biosensor applications. The image was captured at 50,000× magnification with an accelerating voltage of 7.50 kV. The nanoparticles exhibit a spherical morphology with rough surface textures and a tendency to aggregate due to nanoscale van der Waals interactions. The particle population appears uniformly distributed, supporting the DLS-based findings of a monodisperse system. Aggregation clusters are also visible, which is typical for nanomaterials lacking surface stabilization. The scale bar corresponds to 2 µm.

Scanning Electron Microscopy (SEM) (Apreo S model-FEG, Thermo Scientific, Waltham, MA, USA) analysis revealed spherical nanoparticles with rough surfaces, prone to aggregation due to nanoscale van der Waals interactions (see Figure 2). Size distribution measurements from random SEM fields showed an average particle diameter of 185±52 nm (n=100). Hydrodynamic size distribution analysis of the MNPs was previously performed using dynamic light scattering (DLS) prior to any surface modification steps. As reported in our earlier study conducted by Gürcan et al., 2024, the extracted MNPs exhibited an average hydrodynamic diameter of 228±50 nm (n=3) in deionized water, with a polydispersity index (PDI) of 0.12±0.03, confirming a monodisperse and uniform particle population suitable for biosensing applications [33]. Furthermore, the surface chemistry of MNPs was characterized using ATR-FTIR spectroscopy, which revealed prominent peaks corresponding to –OH and –NH stretching (approximately 3303 cm^−1^), aliphatic C–H stretching (2926 cm^−1^), aromatic C–N vibration (1550 cm^−1^), and pyrrole ring deformation (1329 cm^−1^). These bands confirmed the presence of carboxylic acid, phenolic, and aromatic amino groups, with surface amines in particular providing reactive sites for covalent bioreceptor immobilization [18,33,36,37,38]. Notably, these functional groups remained intact throughout the subsequent modification processes, enabling effective antibody conjugation.

### 2.3. Detection of Sensor Regions Using Segmentation Methods

Segmentation refers to the automated identification and separation of important regions within an image. In our case, it helps detect the sensor areas to enable accurate measurement of color changes. To evaluate the performance of the segmentation methods used in this study, two commonly used metrics were employed: Intersection over Union (IoU) and Dice Similarity Coefficient. IoU quantifies the overlap between the predicted and actual areas. It is calculated by dividing the area of overlap by the total area covered by both. The IoU value ranges from 0 to 1, where 1 indicates a perfect match:

(1) $$\mathrm{IoU}=\frac{\left|A\cap B\right|}{\left|A\cup B\right|}$$

Dice Score is another commonly used metric for evaluating overlap. It uses a slightly different formula that places greater emphasis on the intersecting region. Like IoU, the Dice Score also ranges from 0 to 1, and a value of 1 indicates a perfect correspondence between the predicted and ground truth regions:

(2) $$\mathrm{Dice}\;\mathrm{Score}=\frac{2\;\cdot \;|A\cap B|}{\left|A|+|B\right|}$$

Here, *A* and *B* are the predicted and actual segmentation masks. These two metrics are widely used in image segmentation, especially in medical and scientific applications, to check if the model is performing well.

### 2.4. Data Collection and Labeling

To evaluate the performance of a paper-based colorimetric biosensor functionalized with anti-CA19-9-coated melanin nanoparticles, images were captured under laboratory conditions using a smartphone camera. Each image included both the control (unaltered surface) and the target (biosensor surface exposed to CA19-9), ensuring minimal impact from external factors such as lighting variations and camera angle. By capturing the control and target regions side by side, the analysis became more reliable, as any environmental influences would affect both regions equally. This setup enabled the accurate quantification of colorimetric changes in response to varying CA19-9 concentrations.

A total of 100 images were collected, with each image containing both the control and target regions. This resulted in a total of 200 segmented biosensor regions for analysis. Histogram-based feature extraction was performed on each region to quantify color intensity variations. The histograms provided a detailed distribution of color changes, which were then processed to compute statistical features. These features facilitated an objective and quantitative evaluation of the biosensor’s response to different biomarker concentrations.

**Figure 3:**
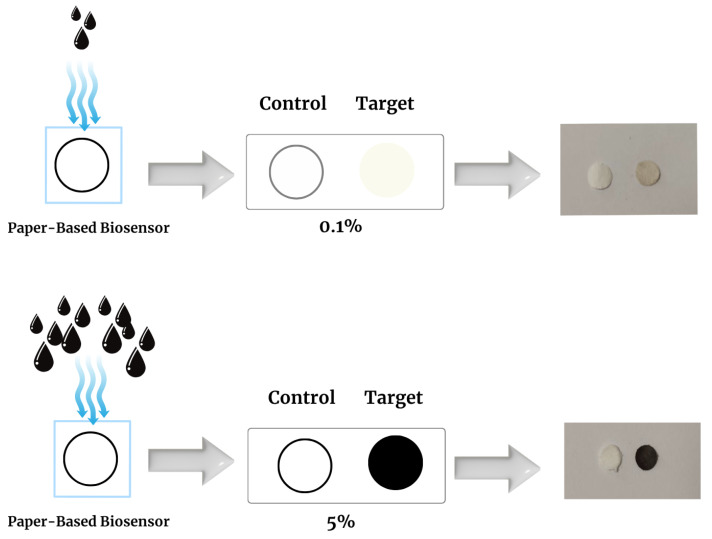
Visual demonstration of colorimetric response of the paper-based biosensor at two different biomarker concentrations (0.1% and 5%). In both cases, the control zones remain visually unchanged, confirming the stability and reliability of the reference region. At 0.1% concentration, limited biomarker binding induces a slight color shift in the target zone due to minimal melanin nanoparticle accumulation. In contrast, the 5% concentration leads to a strong visual signal, indicating enhanced binding and increased melanin nanoparticle formation. This concentration-dependent color variation enables semi-quantitative detection of biomarker levels.

Throughout the study, biosensors were exposed to CA 19-9 concentrations of 0.0125%, 0.025%, 0.05%, 0.075%, 0.1%, 1%, 2%, 3%, 4%, and 5%. The resulting images captured the colorimetric shifts associated with each concentration level. Figure 3 illustrates sample images corresponding to 0.1% and 5% CA 19-9 concentrations, where the right side of each image represents the target region and the left side represents the control. This experimental design offers a consistent and systematic approach for evaluating the biosensor’s performance under different conditions, ensuring the reliability and reproducibility of the analysis.

The Roboflow labeling tool [39] was utilized for data annotation in the next stage of the study. The prepared dataset, consisting of images with varying melanin concentration levels, was uploaded to the platform. Subsequently, the sensor regions in the images were manually annotated to create the ground truth data. The annotated dataset used in this study has been publicly released to support future research and benchmarking efforts [40].

### 2.5. Feature Extraction Methodology

Once the segmented control and target regions were separated, the color differences between these areas provided valuable information on biomarker concentration. Therefore, various color-based features were extracted from these two sensor regions to quantify the changes effectively.

The feature extraction process in this study relied on histogram-based analysis of the control and target regions. The control region served as a fixed reference point representing the baseline (unaltered) surface, enhancing the reliability of the analysis and reducing variability caused by image capture conditions. The target region represented the area exposed to varying CA19-9 biomarker concentrations.

A total of 100 images were analyzed, with control and target regions examined in each, resulting in 200 segmented sensor regions. Histograms from these regions provided a detailed representation of color intensity variations. These histograms were systematically recorded, and statistical features were computed based on the intensity distributions of each region. The extracted features are listed in Table 1. Feature differences between the control and target regions served as input for regression algorithms to predict CA19-9 concentration. This approach enabled a quantitative and unbiased assessment of color intensity changes caused by different CA19-9 concentrations, allowing for robust and interpretable analysis. To utilize the extracted feature differences between control and target regions for concentration estimation, machine learning regression models were employed to capture the underlying relationship.

### 2.6. Prediction of CA 19-9 Concentration Using Regression Algorithms

We used conventional regression models to estimate the CA 19-9 concentration from the extracted sensor features. Regression analysis was used to model the relationship between the dependent variable (CA 19-9 concentration) and the independent features, allowing for accurate and continuous estimation.

Among the regression techniques evaluated, Linear Regression (LR) was first employed as a baseline model due to its simplicity and interpretability. It assumes a linear relationship between the input features and CA 19-9 concentration. However, LR can be sensitive to outliers and may overfit the data, particularly when the dataset is small. To mitigate these limitations, Lasso Regression was applied. It incorporates L1 regularization, which drives some feature coefficients to zero, thereby simplifying the model and performing implicit feature selection—a valuable property when working with limited or noisy datasets.

In addition to linear models, Decision Tree (DT) regression was applied to capture potential non-linear relationships. While DTs are flexible, they can easily overfit small datasets unless properly regularized or pruned. Support Vector Regression (SVR) was also considered for its ability to model non-linear relationships through kernel-based transformations. It is known to be effective in handling high-dimensional feature spaces, although its performance can be influenced by data distribution and sensitivity to feature scaling—particularly in small datasets.

To explore instance-based learning, K-Nearest Neighbors (KNN) regression was implemented. KNN estimates target values based on feature similarity to nearby examples. Although effective for capturing local patterns, its performance can be unstable with sparse or limited datasets and becomes computationally expensive as dataset size increases.

To enhance predictive accuracy and robustness, ensemble learning methods were employed. Random Forest (RF), an ensemble of Decision Trees trained via bootstrap aggregation (bagging), effectively reduced variance and mitigated individual tree biases. Gradient Boosting Machines (GBMs) were also utilized, iteratively refining predictions by minimizing residual errors in a stage-wise fashion. Furthermore, XGBoost, an optimized implementation of gradient boosting with regularization, was employed due to its efficiency and superior predictive performance on structured data.

Overall, the ensemble-based approaches, particularly XGBoost and GBM, demonstrated the most reliable performance in CA 19-9 concentration prediction, outperforming conventional regression techniques in terms of accuracy and generalization.

To quantitatively assess the performance of the regression models in predicting CA 19-9 concentration, multiple evaluation metrics were employed. These metrics provided insights into predictive accuracy, error magnitude, and overall model reliability. To evaluate how well each model captured the relationship between the extracted features and CA 19-9 concentration, several statistical metrics were computed, beginning with the $R^{2}$ score.

The $R^{2}$ score, also known as the coefficient of determination, is a statistical measure that indicates how well the regression predictions align with actual values. It is defined as

(3) $$R^{2}=1-\frac{\sum_{i=1}^{L}{\left(y_{i}-{\hat{y}}_{i}\right)}^{2}}{\sum_{i=1}^{L}{\left(y_{i}-\bar{y}\right)}^{2}},\;\bar{y}=\frac{1}{L}\sum_{i=1}^{L}y_{i}$$

where $y_{i}$ represents the actual values, ${\hat{y}}_{i}$ denotes the predicted values, *L* is the total number of samples, and $\bar{y}$ is the mean of the actual values. $R^{2}$ ranges from 0 to 1, where values closer to 1 indicate that the model explains most of the variance in the data, whereas lower values suggest weaker predictive performance. While the $R^{2}$ score provides a measure of how well the model explains variance, it does not convey the actual magnitude of prediction errors. To address this, the Mean Squared Error (MSE) was used to quantify the average squared difference between predicted and actual values.

The Mean Squared Error (MSE) measures the average squared difference between predicted and actual values:

(4) $$MSE=\frac{1}{L}\sum_{i=1}^{L}{\left(y_{i}-{\hat{y}}_{i}\right)}^{2}$$

MSE emphasizes large errors due to squaring, making it useful for identifying models with significant deviations. However, it does not show how big the errors are compared to the true values. Therefore, the Mean Absolute Percentage Error (MAPE) was used as a complementary metric.

MAPE quantifies prediction accuracy by computing the mean absolute percentage difference between actual and predicted values:

(5) $$MAPE=\frac{1}{L}\sum_{i=1}^{L}\left|\frac{y_{i}-{\hat{y}}_{i}}{y_{i}}\right|\times 100$$

MAPE is particularly relevant for CA19-9 concentration predictions, as it normalizes errors relative to the actual values, thereby enhancing interpretability.

By analyzing all evaluation metrics together, the most appropriate regression model for CA19-9 concentration prediction was identified, ensuring both high accuracy and robustness.

## 3. Results

Traditional segmentation methods, including thresholding, edge detection, and clustering-based algorithms, rely on low-level image features such as color, intensity, and texture. While computationally efficient, these methods often fall short when applied to biosensor images, which are typically characterized by non-uniform illumination, subtle color gradients, and visual noise resulting from environmental factors. For example, thresholding techniques struggle to capture gradual chromatic transitions induced by biochemical reactions, and edge detection approaches often fail to delineate diffuse or fuzzy boundaries. Similarly, clustering algorithms like k-means depend on predefined similarity measures, which may not sufficiently capture the complex visual characteristics of biosensor surfaces.

In contrast, deep learning-based segmentation approaches have demonstrated superior performance due to their ability to learn high-level, abstract feature representations from large datasets. These models can automatically adapt to variations in sensor geometry, lighting conditions, and background noise, thereby eliminating the need for manual parameter tuning. In this study, we selected and evaluated three widely adopted deep learning models with complementary strengths: U-Net [41], YOLOv8 [42], and Detectron2 [43]. U-Net, with its encoder–decoder architecture and skip connections, has been extensively used in biomedical image segmentation and is particularly effective in scenarios requiring precise pixel-level delineation. YOLOv8 and Detectron2, on the other hand, are state-of-the-art object detection and segmentation frameworks capable of real-time processing, making them well-suited for high-throughput biosensor applications.

**Figure 4:**
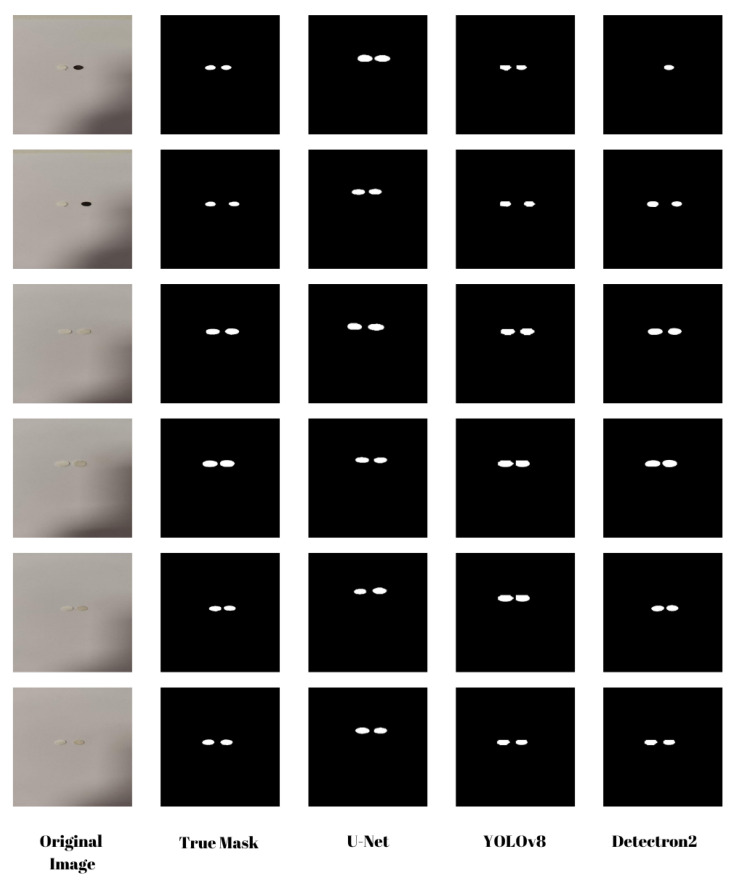
Comparison of segmentation results for different deep learning models. The first column shows the original biosensor images, and the second column presents the corresponding ground truth masks. The third to fifth columns display the predicted masks generated by U-Net, YOLOv8, and Detectron2, respectively. While the overall segmentation performance results appear visually similar, small differences in the detected target regions can be observed. Notably, in the first example, Detectron2 fails to identify the control biosensor region, highlighting a critical discrepancy. These visual results complement the quantitative evaluation provided in Table 2.

Accurate segmentation of biosensor images is essential for extracting meaningful features and ensuring precise biomarker quantification. In this work, we systematically evaluated the performance of U-Net, YOLOv8, and Detectron2 on a curated biosensor image dataset (see Figure 4). Each model was trained and tested on biosensor images, and their segmentation performance was assessed using two widely used evaluation metrics: Dice Coefficient and Intersection over Union (IoU).

The segmentation results for the training and test datasets are summarized in Table 2.

The results indicate that U-Net achieved the highest segmentation accuracy, outperforming both YOLOv8 and Detectron2 with a Dice Coefficient of 0.9483 and an IoU of 0.9025 on the test set. Despite the relatively simple structure of the biosensor images, consisting of two circular regions, U-Net’s encoder–decoder architecture with skip connections allowed for accurate boundary detection and consistent segmentation performance across samples.

YOLOv8 also performed well, achieving a Dice score of 0.9225 and an IoU of 0.8565 on the test set. Although it is computationally efficient and suitable for real-time segmentation tasks, it showed slightly lower precision compared to U-Net, especially in complex image regions requiring fine boundary delineation.

Detectron2, although stable during training, exhibited the lowest segmentation performance among the three models, with a Dice Coefficient of 0.8025 and an IoU of 0.8022 on the test set. Its higher standard deviation indicates that the model encountered difficulties in generalizing to more complex or diverse test images. Performance improvements may be achieved through further hyperparameter tuning, data augmentation, or fine-tuning on a larger, more diverse dataset.

Overall, these findings highlight U-Net’s effectiveness for biosensor segmentation tasks, particularly when high precision is required. YOLOv8 remains a viable alternative for applications where computational efficiency is a priority, while Detectron2 may benefit from additional optimization efforts to enhance its segmentation accuracy.

**Figure 5:**
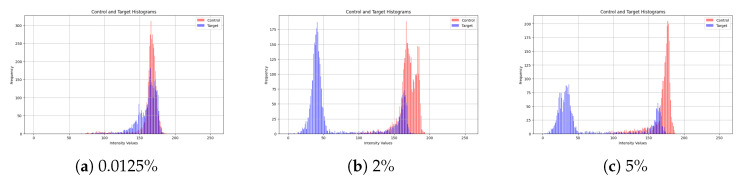
Intensity histograms of control and target regions at varying CA19-9 concentrations. Histograms show the distribution of pixel intensity values for the control (blue) and target (red) regions of the biosensors at three CA19-9 concentrations: (**a**) 0.0125%, (**b**) 2%, and (**c**) 5%. At low concentration (0.0125%), there is significant overlap between the histograms of control and target regions, indicating a subtle colorimetric change. As the biomarker concentration increases, particularly at 2% and 5%, the histograms show progressively better separation, with minimal overlap at 5%. This reflects the biosensor’s enhanced ability to distinguish target responses due to higher levels of melanin nanoparticle binding, resulting in a pronounced darkening of the target region. The visual separation between histograms confirms the sensor’s potential for semi-quantitative detection of CA19-9 levels.

To evaluate the impact of CA19-9 concentration on sensor response, we generated intensity histograms for multiple concentration levels. While our study includes 10 different concentration values, Figure 5 presents a representative subset, showcasing the results for 0.0125%, 2%, and 5%. These histograms illustrate the intensity distributions of the control and target sensors, where the control sensor remains unchanged while the target sensor undergoes a colorimetric shift due to CA19-9 binding.

At low concentrations (0.0125%), the color change is minimal, resulting in substantial overlap between the control and target histograms. This overlap indicates a weak sensor response at low biomarker levels, which makes accurate differentiation more difficult.

As the concentration increases (2%), a noticeable shift in the intensity distributions is observed. The separation between the control and target histograms becomes more evident, indicating a stronger sensor response.

At higher concentrations (5%), the histograms show clear separation, with minimal overlap. This confirms a pronounced colorimetric shift in the sensor as CA19-9 concentration increases, facilitating more accurate detection.

Overall, the results indicate that higher CA19-9 concentrations produce more distinguishable color changes, supporting the feasibility of intensity-based analysis for automated concentration estimation.

As a next step, statistical features were extracted from these histograms as presented in Table 1. These features, which capture the distributional differences between the control and target histograms, were then used as inputs for regression models to predict CA19-9 concentration levels.

To model the relationship between sensor responses and CA19-9 concentration, multiple regression algorithms were implemented and evaluated, including XGBoost, Gradient Boosting, Random Forest, Decision Tree, K-Nearest Neighbors (KNN), Support Vector Regression (SVR), Linear Regression, and Lasso Regression. Model performance was assessed using three key evaluation metrics: Mean Squared Error (MSE), Mean Absolute Percentage Error (MAPE), and the Coefficient of Determination (R^2^), which collectively reflect prediction accuracy, error magnitude, and the proportion of variance explained. Table 3 presents the evaluation metrics for each regression model.

The results indicate that XGBoost and Gradient Boosting were the most effective models, achieving the best balance between prediction accuracy and error minimization. Among all models, XGBoost demonstrated the lowest MAPE (17.37) and MSE (0.328865), along with an R^2^ score of 0.8894, making it the most reliable predictor for CA19-9 concentration. Gradient Boosting performed comparably well, with an MSE of 0.547949 and an R^2^ score of 0.815767, explaining 81.6% of the variance in the dataset.

The Random Forest model also performed well, achieving an R^2^ score of 0.8810 and a relatively low MSE of 0.353841, highlighting its potential as a robust alternative to XGBoost and Gradient Boosting. However, its MAPE (19.60) was slightly higher, indicating greater relative prediction errors.

The Decision Tree model, while straightforward and interpretable, exhibited higher error rates (MSE: 1.000094) and lower variance explanation (R^2^: 0.6637), suggesting it is less suitable for precise predictions.

The Support Vector Regression (SVR) model performed poorly, with an R^2^ score of only 0.6944 and an excessively high MAPE of 134.68, indicating poor generalization to new data. These results suggest that SVR is not well-suited for this dataset without substantial hyperparameter optimization.

Linear models, including Linear Regression and Lasso Regression, yielded the weakest performance overall. Despite Linear Regression achieving a high R^2^ score of 0.8977, its MAPE was extremely high (300.88), underscoring its lack of predictive reliability. Lasso Regression performed even worse, with an MSE of 0.917572 and an R^2^ score of 0.6915, rendering it unsuitable for practical use.

Based on these findings, XGBoost and Gradient Boosting emerged as the most accurate and reliable regression models for predicting CA19-9 concentration. Random Forest also performed well and serves as a robust alternative. Lower-performing models such as Decision Tree, KNN, and SVR exhibited limited generalization capability, while Linear and Lasso Regression showed excessively high percentage errors, making them impractical for this application.

These results suggest that ensemble-based methods (XGBoost, Gradient Boosting, and Random Forest) are best suited for CA19-9 concentration prediction due to their ability to capture non-linear relationships and reduce overfitting through bagging and boosting techniques. In particular, their robustness against noise and strong generalization capability make them well-suited for modeling complex biomarker–feature interactions in colorimetric biosensing applications.

**Figure 6:**
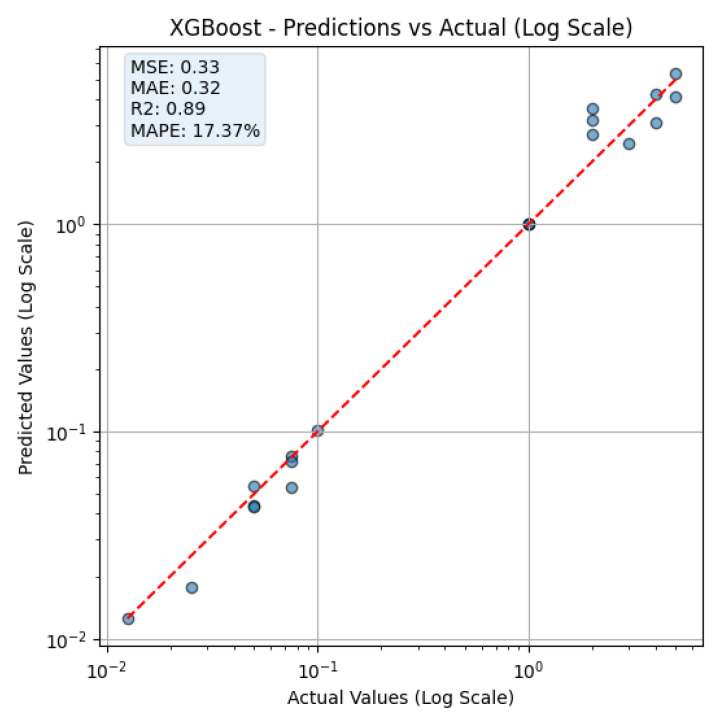
Prediction performance of the XGBoost regression model. The plot compares predicted versus actual CA19-9 values on a logarithmic scale. Each point represents a test sample, and the red dashed line indicates the ideal 1:1 prediction. The model achieved an $R^{2}$ of 0.89, an MSE of 0.33, an MAE of 0.32, and a MAPE of 17.37%, demonstrating strong predictive performance. While the model shows high accuracy in the clinically relevant low-concentration range, deviations at higher concentrations suggest possible sensor saturation, indicating that further tuning may enhance performance in this region.

Figure 6 illustrates the prediction performance of the XGBoost model, comparing the actual CA19-9 concentrations with the predicted values on a logarithmic scale. The figure is shown on a log–log scale to properly visualize low concentration predictions. The apparent gaps between the values result from logarithmic spacing, not from data omission. The red dashed line represents the ideal reference line where predicted values equal actual concentrations (i.e., y=x). Data points closer to this line indicate better prediction accuracy.

At low concentrations, the model accurately predicts CA19-9 levels, which is particularly crucial for early detection of pancreatic cancer. Accurate estimation in this range enhances the potential for timely intervention and diagnosis.

However, at higher concentrations, the prediction error tends to increase. This phenomenon can be attributed to sensor saturation, where beyond a certain CA19-9 level, no significant additional color change is observed. As a result, the extracted histogram features become less sensitive to increasing biomarker levels, limiting the model’s ability to distinguish among very high concentrations accurately.

These findings indicate that, while the XGBoost model provides high accuracy in the clinically relevant low concentration range, further optimization may be required to account for sensor saturation effects at higher concentrations.

## 4. Discussion

This study presents a machine learning-based method for analyzing color changes in a paper-based biosensor that uses natural melanin nanoparticles (MNPs) and anti-CA19-9 antibodies. Our goal was to create a simple, eco-friendly, and smartphone-compatible platform for detecting CA19-9, a biomarker commonly used in pancreatic cancer monitoring. For image segmentation, the U-Net model outperformed other deep learning models such as YOLOv8 and Detectron2. U-Net achieved the highest Dice score and Intersection over Union (IoU), making it the most accurate in detecting biosensor areas. While YOLOv8 was faster and more computationally efficient, it was slightly less precise. Detectron2 showed the lowest performance in our setup, possibly due to the limited size and simplicity of the dataset. In terms of concentration prediction, ensemble models like XGBoost and Gradient Boosting performed the best. XGBoost achieved the lowest Mean Absolute Percentage Error (MAPE) and Mean Squared Error (MSE), and the highest R^2^ score, indicating strong predictive accuracy. Traditional models such as Linear Regression and Support Vector Regression performed worse, with much higher MAPE values, making them less suitable for this application.

Compared to earlier work using traditional PADs with metallic nanoparticles and features such as RGB or HSV histograms [44], our method combines both statistical features and deep learning-based image segmentation, resulting in improved robustness and accuracy. Also, while prior studies like Khanal et al. [44] focused on food and environmental analytes (e.g., protein, iron, and thiocyanate), our system targets a clinically important cancer biomarker (CA19-9), demonstrating that such platforms can be extended to medical diagnostics. Moreover, the biosensor uses melanin nanoparticles, which offer an environmentally friendly alternative to metal-based nanomaterials like gold nanoparticles (AuNPs). While AuNPs are widely used due to their optical properties [7,8], they pose concerns regarding toxicity and sustainability [10,11,13]. MNPs are biodegradable, safer, and still provide effective broadband absorbance for colorimetric detection [16,24]. However, the study has several limitations. First, the dataset consists of only 100 images, which may affect the model’s ability to generalize to new samples. Second, although our image processing pipeline compensates for lighting and angle variations using a control region, it still relies on smartphone camera quality and ambient conditions. Third, the study focused on image-based analysis using pre-applied concentrations of CA19-9, without real-time detection in a fluidic environment. Despite these limitations, the system shows strong potential for point-of-care (POC) testing. Unlike earlier works that required controlled imaging conditions [29,30], our system can analyze images captured in uncontrolled or variable environments. Furthermore, the smartphone-based design makes the platform portable and low-cost, enhancing its suitability for use in remote or resource-limited areas.

Future studies will focus on expanding the dataset, testing in varied lighting conditions, and developing a real-time mobile application. Recent advances in colorimetric nanoparticle systems, such as ferrocene-mediated hydrogels for rapid pH monitoring [45] and portable platforms for biomarker analysis under various conditions [46], support the adaptability of such technologies across biosensing and environmental domains. Moreover, since melanin nanoparticles are quite functional, the same structure can be adapted for other diseases or targets. By combining sustainable nanomaterials with AI-based analysis, this study contributes to the development of next-generation diagnostic tools that are cost effective, reliable, and easy to use in both clinical and field settings.

## 5. Conclusions

In conclusion, an AI-assisted MNP-based paper analytical device (PAD) platform that integrates Sepia-derived melanin nanoparticles, a smartphone-compatible interface, and machine learning-based image analysis for CA19-9 detection is developed. In this study, three key innovations are demonstrated. First, the use of biocompatible and biodegradable melanin nanoparticles offers a sustainable alternative to metal-based nanomaterials while maintaining strong optical responsiveness for colorimetric biosensing. Second, the integration of segmentation and regression models enables the accurate quantification of colorimetric changes related to varying CA19-9 concentrations. Lastly, the platform achieves portable, cost-effective and rapid biomarker monitoring, providing a proof-of-concept for future point-of-care diagnostic tools. With further development such as larger datasets, real-time detection, and fluidic integration, this system has the potential to be adapted to broader clinical and environmental biosensing applications. 

## Figures and Tables

**Table 1:** Summary of intensity-based image features.

Feature	Definition	Formula
Minimum Intensity	Lowest pixel intensity, useful for identifying dark areas and segmentation.	$\mathrm{Min}\left(I\right)=\min\left(I_{1},I_{2},\ldots ,I_{n}\right)$
Maximum Intensity	Highest pixel intensity, essential for brightness-based segmentation.	$\mathrm{Max}\left(I\right)=\max\left(I_{1},I_{2},\ldots ,I_{n}\right)$
Average Intensity	Arithmetic mean of all pixel intensities, representing overall brightness.	$Average\left(I\right)=\frac{1}{n}\sum_{i=1}^{n}I_{i}$
Standard Deviation	Measures intensity variation, indicating contrast and texture.	$\sigma =\sqrt{\frac{1}{n}\sum_{i=1}^{n}{\left(I_{i}-\mu \right)}^{2}}$
Median Intensity	Middle value of sorted intensities, robust against noise and outliers.	$Median\left(I\right)=\mathrm{Middle}\;\mathrm{value}\;\mathrm{of}\;\mathrm{sorted}\;I_{1},I_{2},\ldots ,I_{n}$
Contrast	Difference between maximum and minimum intensities, measuring intensity variation.	Contrast(I)=Max(I)−Min(I)
Entropy	Measures complexity based on the probability distribution of intensities.	$Entropy\left(I\right)=-\sum_{i=1}^{L}p\left(I_{i}\right)\log\left(p\left(I_{i}\right)\right)$
Uniformity	Evaluates the evenness of intensity distribution, indicating smoothness or texture.	$Uniformity\left(I\right)=\sum_{i=1}^{L}p{\left(I_{i}\right)}^{2}$

**Table 2:** Segmentation performance of U-Net, YOLOv8, and Detectron2.

Model	Dice Coefficient (Test Set)	IoU (Test Set)
U-Net	0.9483 ± 0.0223	0.9025 ± 0.0392
YOLOv8	0.9225 ± 0.0230	0.8565 ± 0.0320
Detectron2	0.8025 ± 0.0230	0.8022 ± 0.0320

**Table 3:** Performance comparison of regression models.

Model	MSE	R^2^	MAPE
XGBoost	0.328865	0.889428	17.373331
Gradient Boosting	0.547949	0.815767	32.059490
Random Forest	0.353841	0.881031	19.603958
Decision Tree	1.000094	0.663747	20.583333
K-Nearest Neighbors	0.901234	0.310550	34.575000
Support Vector Regression	0.908822	0.694434	134.681314
Linear Regression	0.304255	0.897703	300.881976
Lasso Regression	0.917572	0.691492	245.013192

## Data Availability

The dataset generated and analyzed during this study is publicly available at the following repository: Karademir, T., Kaleli-Can, G., & Köktürk-Güzel, B.E. (2025). Image Dataset of Paper-Based Biosensor for CA19-9 Detection Using Melanin Nanoparticles: Machine Learning and Deep Learning Analysis for Pancreatic Cancer Biomarker Monitoring. https://doi.org/10.17632/k3yvcw2v5b.1.
